# Multiple-responsive shape memory polyacrylonitrile/graphene nanocomposites with rapid self-healing and recycling properties†

**DOI:** 10.1039/c7ra11484b

**Published:** 2018-01-03

**Authors:** Chenting Cai, Yue Zhang, Mei Li, Yan Chen, Rongchun Zhang, Xiaoliang Wang, Qiang Wu, Tiehong Chen, Pingchuan Sun

**Affiliations:** Key Laboratory of Functional Polymer Materials of Ministry of Education, College of Chemistry, Collaborative Innovation Center of Chemical Science and Engineering (Tianjin), Nankai University Tianjin 300071 China spclbh@nankai.edu.cn; Department of Polymer Science and Engineering, Nanjing University Nanjing 210093 China; State Key Laboratory of Medicinal Chemical Biology, Nankai University Tianjin 300071 P. R. China; Institute of New Catalytic Materials Science, School of Materials Science and Engineering, Key Laboratory of Advanced Energy Materials Chemistry (MOE), Nankai University Tianjin 300350 P. R. China

## Abstract

It still remains a great challenge to endow polymer materials with multiple superior material properties by precise molecular design. Herein, we report a Diels–Alder (DA) based crosslinked polyacrylonitrile/graphene nanocomposite (PAN-DA/GR), which has multiple-responsive properties of shape memory, self-healing, and reprocessing in addition to enhanced mechanical properties. The graphene sheets, which are well dispersed in the DA-based crosslinked PAN network, can act as intrinsic localized thermal sources by converting the absorbed external IR/microwave energies into heat, to trigger the glass transition for elasticity-based shape memory properties and retro-DA (rDA) reactions for healing. The incorporation of Diels–Alder bonds also gives the material solid state plasticity through topological network rearrangement, thus leading to versatile shape adaptability. Moreover, both regional shape control and targeted self-healing of the nanocomposites can be simply achieved by IR laser irradiation. Besides, the incorporation of a small amount of graphene can significantly improve the mechanical strength with respect to the DA-based crosslinked PAN. Both DSC and *in situ* variable temperature ^13^C solid-state NMR experiments were used to monitor the reversible DA reactions.

## Abstract 2

A Diels–Alder covalent bond based crosslinked polyacrylonitrile/graphene nanocomposite is reported, which has the multiple-responsive properties of shape memory, self-healing, and reprocessing in addition to enhanced mechanical property.

## Graphical abstract

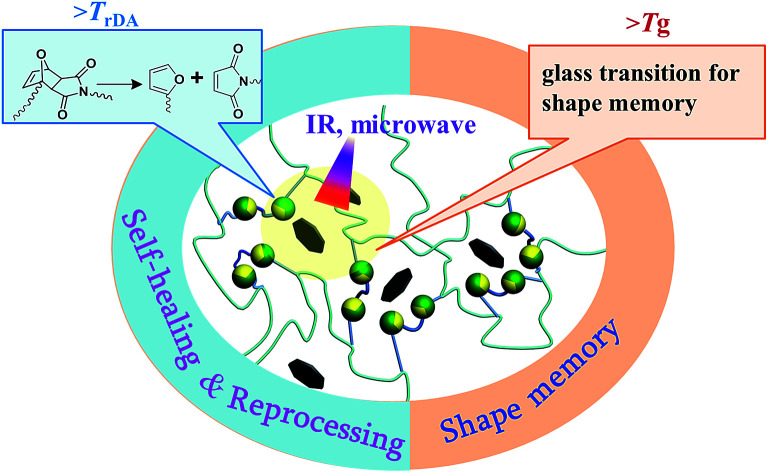

## Introduction

Although significant advances have been achieved for the development and fabrication of high performance polymeric materials in recent decades, it still remains a great challenge to integrate superior material properties, such as shape memory, self-healing and recycling, multiple-responsiveness to external stimuli, into one structure by precise design of molecular architectures.^1–6^ Because of their ability to remember and recover their previous permanent shape under certain stimuli, such as heat or light, shape memory polymers (SMPs) have attracted significant attention in the past few decades.^7–12^ Usually, shape memory properties can be achieved in crosslinked polymers with the appropriate glass transition temperature (*T*_g_) or crystalline domains, where the covalent cross-linkages act as an effective fixed phase and the crystalline domains or glassy chains as the reversible switching phase.^13,14^ However, SMPs generally cannot be reprocessed once synthesized or remended when damaged due to the presence of permanent chemical crosslinking. Inspired by the biological healing function in nature, self-healing polymers have been the focus of recent studies on stimuli-responsive polymer materials,^15–24^ due to their capability of self-healing after suffering from damage or fractures. In particular, reversible crosslinking using dynamic covalent bonds, such as the reversible Diels–Alder (DA) reaction,^25,26^ provides a new strategy to overcome the above difficulty in crosslinked SMPs.^27–29^ However, most of the DA-based SMPs can only be healed or reprocessed by heating-induced retro-DA reaction, which could severely limit their applications in a wide range of circumstances. Therefore, until now, it still remains a great challenge to develop crosslinked SMPs with superior mechanical properties and excellent capability of rapid self-healing and recycling *via* multiple approaches.

**Scheme 1:**
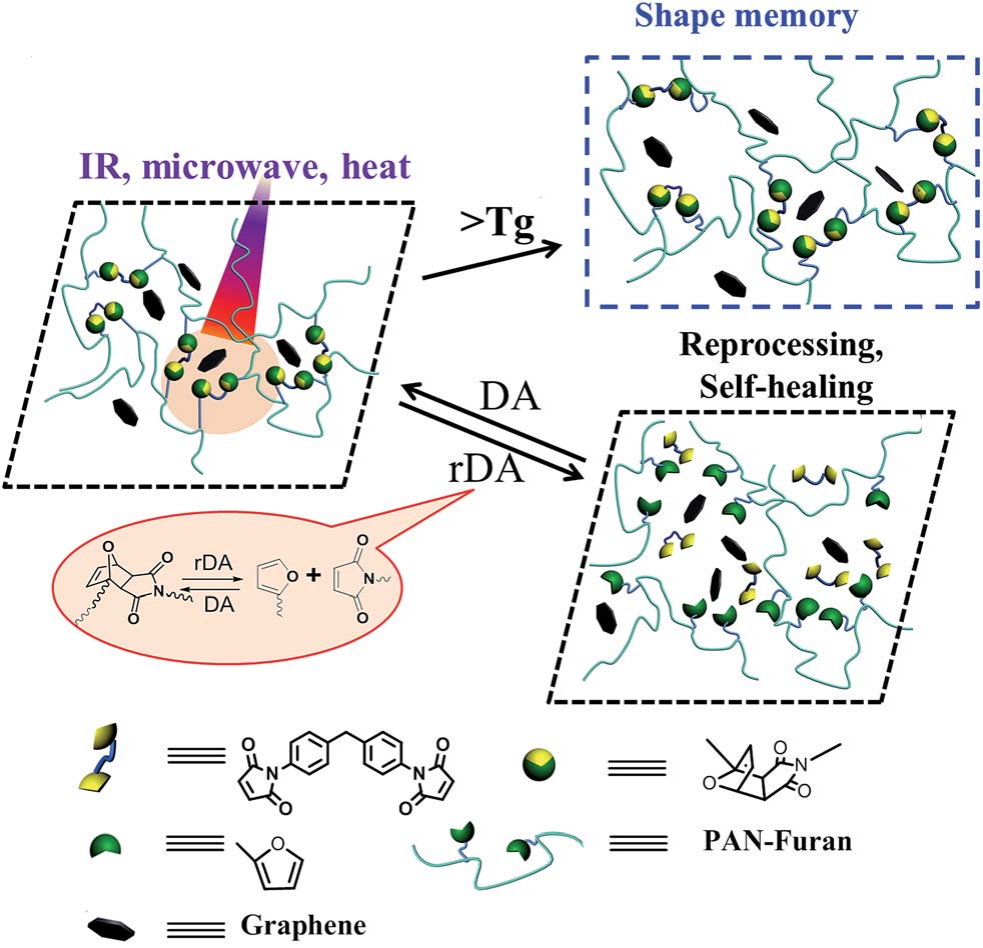
Schematic diagram of the shape memory, healing and reprocessing mechanism by triggering glass transition and rDA reaction in thermally reversible DA-crosslinked PAN-DA/GR nanocomposites *via* multiple approaches including heat, microwave and IR light.

Due to the outstanding mechanical properties of graphene, it has been widely used as a nanofiller for the fabrication of mechanical enhanced nanocomposites.^30–34^ Moreover, graphene can absorb IR and microwave energies and instantly converts them into heat, and thus enable the polymer materials with self-healing property by multiple approaches. Besides, the Diels–Alder bonds can also render the material solid state plasticity through topological network rearrangement at an elevated temperature, and thus leading to versatile shape adaptability. Therefore, it is expected that the combination of graphene and thermo-reversible Diels–Alder (DA) reaction can provide an attractive avenue to prepare novel multiple-responsive nanocomposites with shape memory, self-healing and recycling properties. Recently, we have demonstrated that rapid self-healing and recycling for the epoxy resin (ER) can achieved by simultaneously incorporating thermally reversible DA covalent bonds and graphene into the ER matrix.^35^ Herein, we further report a thermally reversible DA-crosslinked polyacrylonitrile/graphene nanocomposites (PAN-DA/GR) with outstanding mechanical strength as well as self-healing and shape memory properties triggered by multiple approaches. The well-dispersed graphene sheets in proximity to the DA crosslinkages can act as an intrinsic localized thermal source, by converting absorbed energies (IR, microwave, *etc.*) into heat, to induce the polymer glass transition and trigger retro-DA (rDA) reactions. As a result, shape memory capability can be controlled by the glass transition at a temperature beyond *T*_g_, whereas the self-healing/recycling properties can be achieved when the temperature is higher than rDA temperature (*T*_rDA_). Hence, the incorporation of graphene not only improves the mechanical properties with respect to the pristine crosslinked polymer, but also enables controlling the materials properties of self-healing, recycling and shape memory *via* multiple approaches including IR light, microwave and heating, as shown in Scheme 1a. As far as we know, there are few examples about integrating multiple-responsive shape memory and self-healing properties in a single chemical structure. Our work is the first study using microwave and IR to simultaneously realize shape memory, crack healing and reprocessability in one DA cross-linked polymer/graphene nanocomposite.

## Experimental

The schematic procedures for the preparation of PAN-DA/GR nanocomposite is shown in Scheme 1. In order to well separate the *T*_g_ of the polymer nanocomposite from the rDA reaction temperature *T*_rDA_, methyl butylacrylate (BA) (∼15 wt%) and glycidyl methacrylate (GMA) with epoxy group (∼10 wt%) was co-polymerized with acrylonitrile (AN) monomers to obtain PAN-epoxy compounds. Subsequently, PAN-epoxy was dissolved in DMF solvents in together with furfurylmercaptan (2.8 g) and triethylamine to obtain linear PAN-furan for further DA reaction with bismaleimide (BM), *i.e.* PAN-DA compounds. For the PAN-DA/GR nanocomposites, PAN-furan, BM and reduced graphene oxide were both dissolved in NMP solvent for the DA reaction at 60 °C. Four samples, corresponding to a graphene weight fraction of 0, 0.2%, 0.4% and 0.6%, were prepared and named as PAN-DA/GR, PAN-DA/GR-0.2, PAN-DA/GR-0.4, PAN-DA/GR-0.6, respectively. More details about the chemical synthesis and the experimental details about the characterization methods can be found in the ESI.† The well-dispersion of grapheme in the PAN-DA network is clearly demonstrated by the SEM experiment as shown in Fig. S5 in the ESI.†

## Results and discussion

**Scheme 2:**
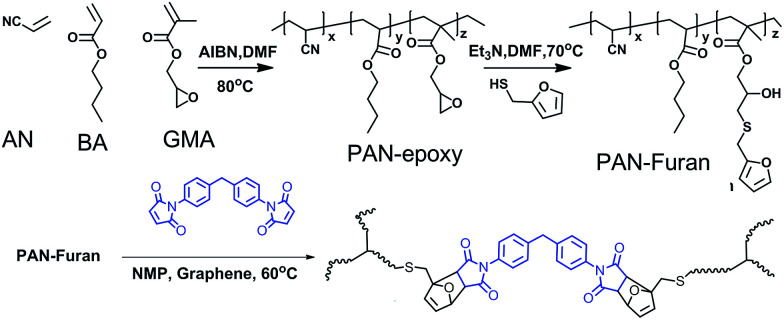
Synthetic route for thermal reversible crosslinked PAN-DA.

Our key ideas of the molecular design shown in Schemes 1 and 2 include: (1) incorporation of reversible DA covalent crosslinks *via* DA reaction between furan pendant groups in linear PAN-furan and the maleimide groups of cross-linker BM. In this way, thermal reversibility, related to recyclability and self-healing ability, can be achieved by the rDA reaction. (2) The monomers BA and GMA were incorporated into linear PAN to adjust the *T*_g_ of the nanocomposite that controls the shape memory property, so that the glass transition is not overlapped with the rDA reactions. In addition, it also greatly enhanced the mechanical toughness of the polymer. (3) The incorporation of a small amount of graphene not only improves the mechanical strength, but also remarkably enhances the efficiency of healing and recycling and enables controlling shape memory property *via* multiple approaches including IR light, microwave and heating due to the multiple-responsive nature of graphene. Besides, regional shape memory property and targeted self-healing can be achieved in response to local IR irradiation. Herein, a multiple-responsive crosslinked SMP with excellent mechanical properties and superior capability of rapid self-healing and reprocessing as well as shape-memory property is well demonstrated.

### Reversible DA reaction revealed by DSC and solid-state ^13^C NMR spectroscopy

**Fig. 1:**
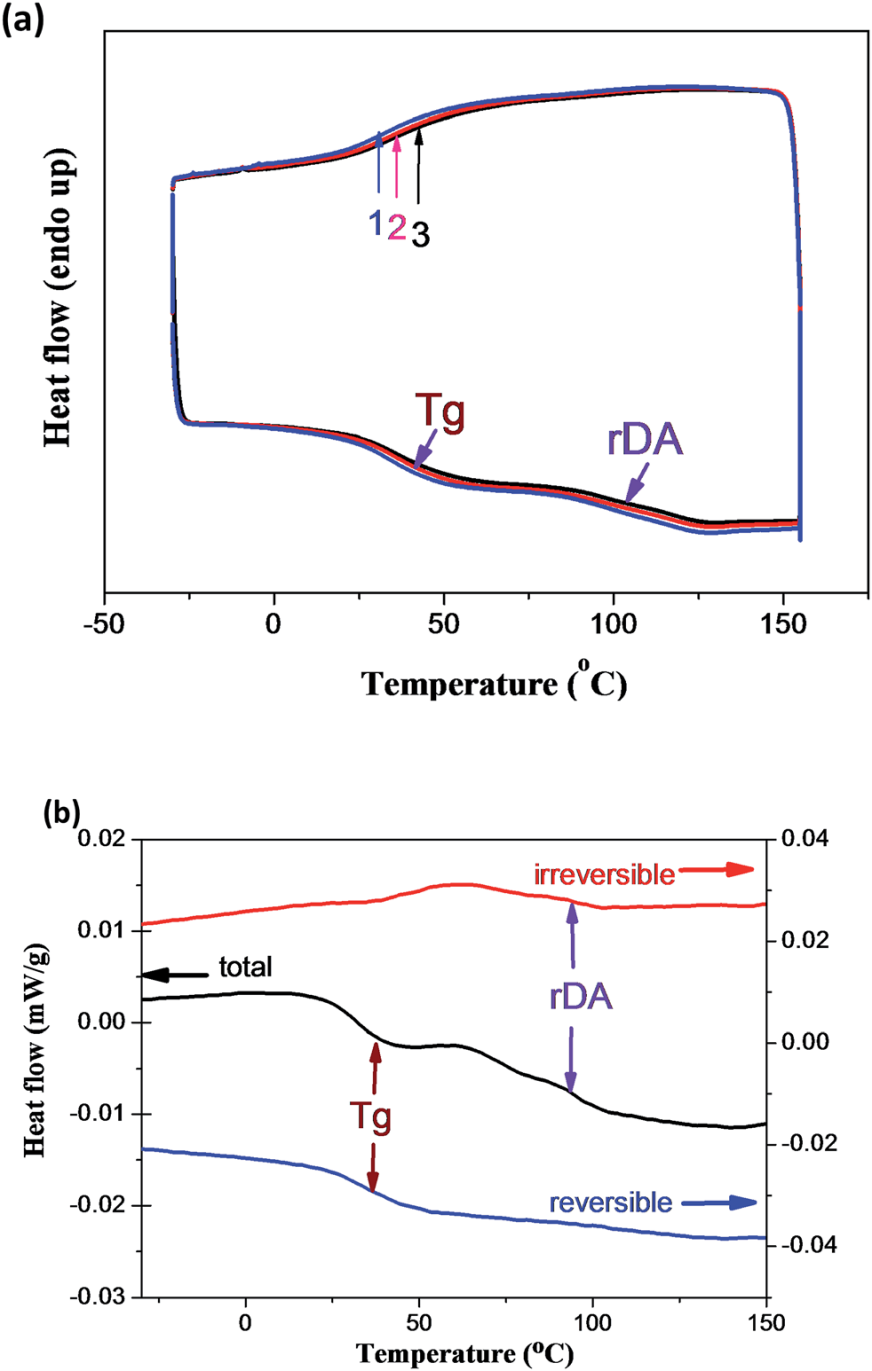
(a) DSC traces of PAN/GR-0.4 in repeated cooling and heating cycles at a rate of 10 °C min^−1^. (b) Temperature modulated DSC (TOPEM) curves of PAN-DA/GR-0.4 sample. The heat flow for the reversible and irreversible thermal transitions, corresponding to the glass transition and retro-Diels–Alder reaction, are well separated.

Repeated DSC cycles and *in situ* variable temperature solid-state ^13^C NMR spectroscopy have been well explored and widely used for the investigation of reversible DA reactions. Fig. 1a showed the enthalpy changes in the repeated DSC cycles, where an endothermic/exothermic transition at about 80–130 °C is clearly observed during the successive heating/cooling cycles. An excellent repeatability of the DSC traces is clearly observed, indicating the complete reconstruction of DA network within a short time scale during the DSC measurements at a heating rate of 10 °C min^−1^. In fact, complete separation of the enthalpy change induced by the glass transition and rDA reaction can be achieved by temperature modulated DSC (TOPEM) experiment, which is a new stochastic temperature-modulated DSC technique introduced by Mettler-Toledo in late 2005 (Fig. 1b).^36^ Obviously, the irreversible heat flow can be ascribed to the rDA reaction, while the reversible one is resulted from the glass transition. The *T*_g_ and *T*_rDA_ determined from the regular DSC cycle are in a good agreement with that obtained from TOPEM experiments. The incorporation of grapheme slightly decrease the *T*_g_, while the *T*_g_ increases with increasing the content of graphene (Fig. S3†).

**Fig. 2:**
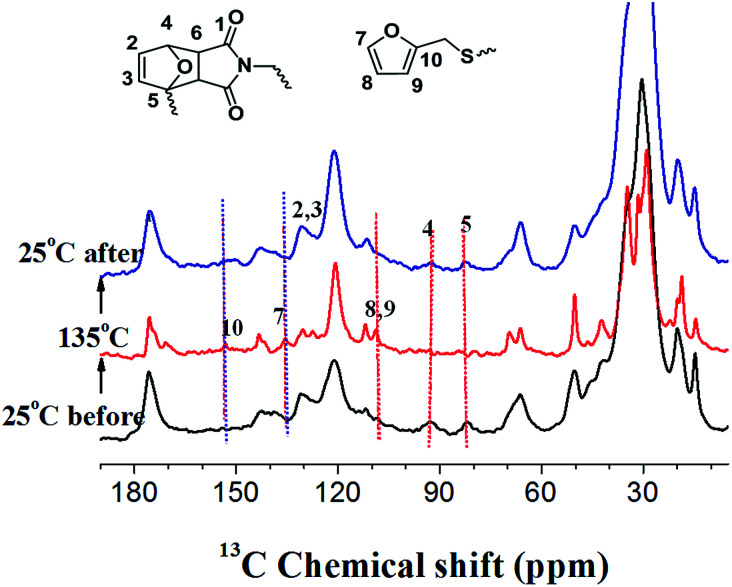
*In situ* variable-temperature ^13^C SSNMR spectra of PAN-DA/GR-0.4 nanocomposite at a thermal cycle of 25 °C (black), 135 °C (red) and 25 °C (blue), respectively. The CPNOE experiment was applied to detect both signals of rigid and mobile components during thermal cycle, and a NOE mixing time of 0.5 s is used under a magic-angle-spinning (MAS) rate of 10 kHz.

*In situ* variable temperature (VT) solid-state ^13^C NMR experiments were further used to demonstrate the reversibility of DA reaction at the molecular level. Due to the enhanced segmental mobility at the high temperature, which will significantly decrease the cross polarization (CP) efficiency, a recently developed novel ^13^C CPNOE technique^37^ was utilized. Thus, the ^13^C signals of rigid components can be enhanced by CP, while that of mobile components can be enhanced by the NOE (nuclear Overhauser effect)-based polarization transfer. Fig. 2 shows the ^13^C CP spectra of PAN-AN/GR-0.4 nanocomposite at a thermal cycle of 25 °C (black), 135 °C (red) and 25 °C (blue), respectively. It is clearly shown that the peaks at 92 and 82 ppm associated with DA-bonds disappeared when temperature was increased from 25 °C to 135 °C; meanwhile, the intensities of small peaks associated with free furan groups (at 108, 135, and 152 ppm) grow significantly. The above results unambiguously confirm the presence of unreacted furan moieties in the system due to the disassociation of DA adducts at the high temperature. When the sample temperature was decreased to 25 °C, the ^13^C spectrum is identical to that obtained before increasing the temperature, indicating that the DA-based crosslinked network was completely reconstructed after the thermal cycle. Because the IR light and microwaves irradiation can be absorbed and converted into heat, the above SSNMR results also indirectly confirm the reversible DA/rDA reactions under IR or microwave irradiation.

### Enhanced mechanical strength

**Fig. 3:**
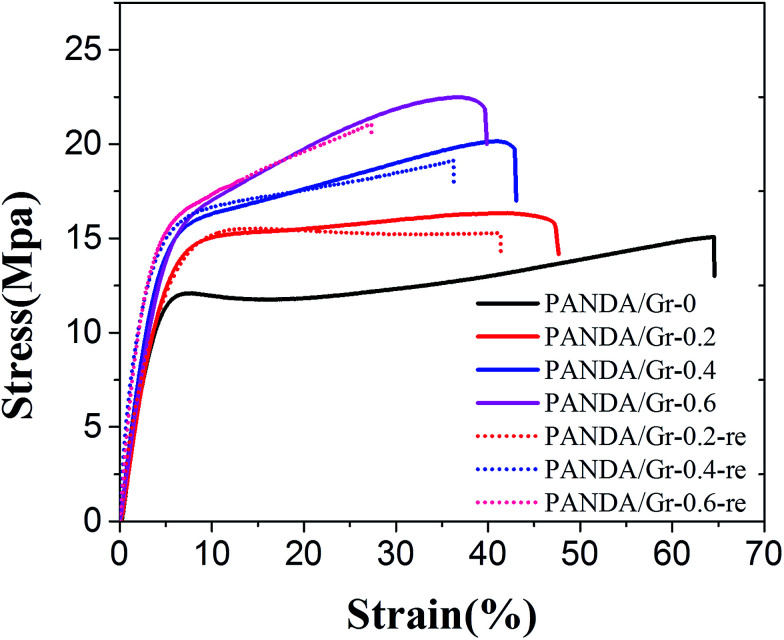
Stress–strain curves of PAN-DA/GR with different graphene content as well as the corresponding recycled samples measured at a strain rate of 5 mm min^−1^ and room temperature.

Graphene is well known as a nanofiller for enhancing the mechanical strength. As shown in Fig. 3 and Table 1, due to the well-dispersed graphene sheets in the PAN-DA matrix, the incorporation of less than 1 wt% graphene greatly enhanced the tensile strength at break and Young's modulus, while the elongation at break is slightly compromised. However, multiple approaches can be applied for self-healing and recycling as well as controlling of shape memory properties due to the incorporation of such small amount of grapheme as discussed below in detail. The tensile strain–stress curves of the recycled samples were also shown in Fig. 3, where the mechanical properties were only slightly compromised with respect to the pristine samples.

**Table 1:** Tensile properties of the PAN-DA/GR with variable graphene content

Samples	Tensile strength (MPa)	Young's modules (MPa)	Elongation at break (%)
PAN-DA/GR-0	14.1 + 1.1	353 + 30	65 + 4
PAN-DA/GR-0.2	15.0 + 1.7	419 + 25	48 + 3
PAN-DA/GR-0.4	18.2 + 1.5	498 + 40	44 + 3
PAN-DA/GR-0.6	22.3 + 1.9	520 + 36	39 + 4

### Shape memory properties

**Fig. 4:**
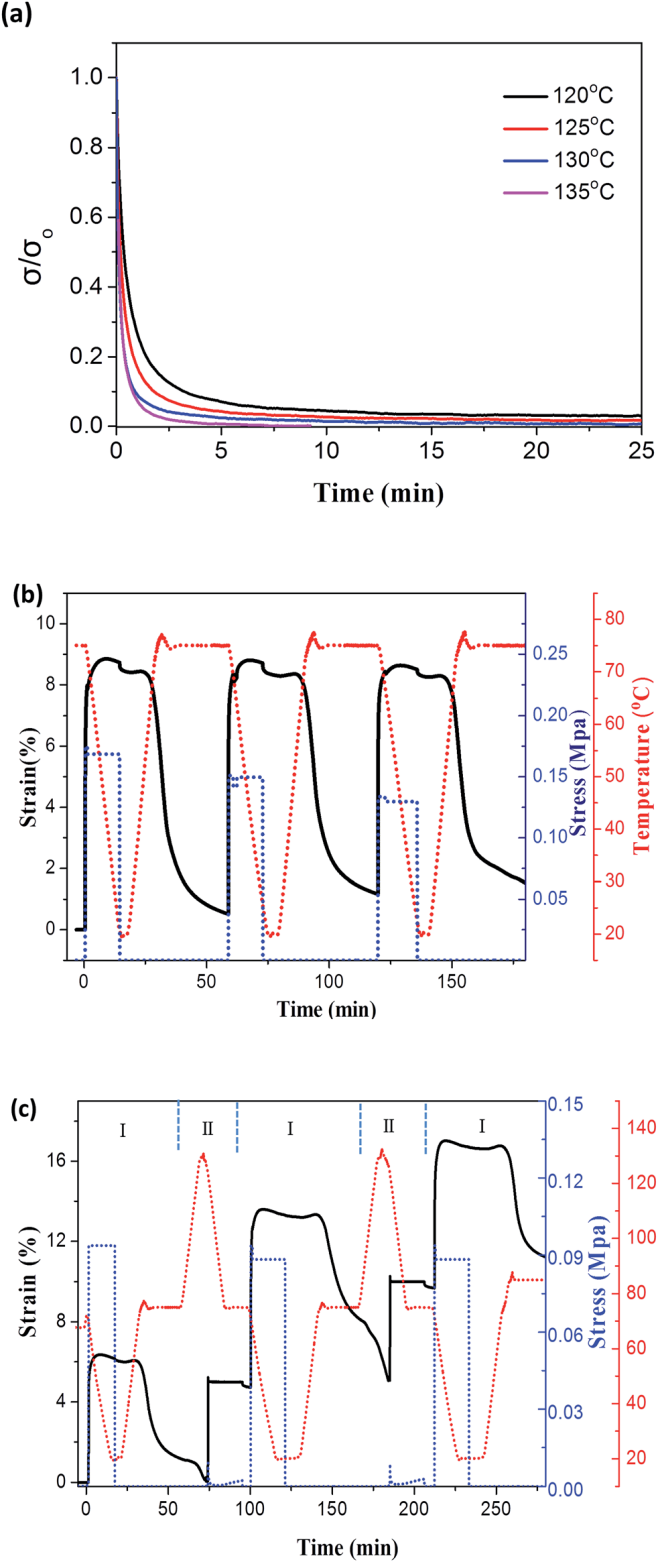
Quantitative demonstration of the shape memory property of PAN-DA/GR-0.4 sample measured by DMA experiments. (a) Stress relaxation at various temperatures crossing *T*_rDA_. (b) Thermomechanical characterization of shape memory cycles. (c) Thermomechanical characterization of alternate elasticity- and plasticity-based reshaping cycles labelled by “I” and “II,” respectively.

Stimuli-responsive shape memory polymers have attracted tremendous attention due to their vast potential in various applications.^38,39^ In general, most shape memory behaviors origin from the elasticity of polymers, where the entropic energies can be stored and released *via* conformational changes of polymer chains. As a result, glass transition has been often used for controlling shape memory properties, where the crosslinkages act as the fixed phase whereas the glassy chains are used as the reversible phase. In contrast, polymer plasticity, where permanent polymer reshaping without melting can be achieved through dynamic bond exchange, has gain dramatic attention in recent years.^40–43^ Particularly, polymer plasticity through DA reaction has been recently examined in the crosslinked epoxy resin network.^27^ Herein, the elasticity and plasticity natures of PAN-DA/GR nanocomposites were well demonstrated by multiple approaches including heat, IR and microwaves, taking the PAN-DA/GR-0.4 as a typical example. Fig. 4a shows the stress relaxation curves of PAN-DA/GR-0.5 at variable temperatures beyond *T*_rDA_. A fixed strain of 15% was initially loaded, and the stresses relaxed faster with increasing the temperature due to the shifted dynamic equilibrium towards rDA reactions. As is clearly seen, complete stress relaxation takes about 5 min at a temperature of 135 °C, which enables faster shape manipulation. Therefore, below we will explore the dynamic bond-based plasticity of PAN-DA/GR-0.4 sample at 135 °C. Quantitative demonstration of the shape-memory property of PAN-DA/GR-0.4 was measured under a stress-controlled mode with identical deformation and recovery temperatures of 75 °C in consecutive shape memory cycles shown in Fig. 4b. In each cycle, the shape fixity ratio and shape recovery ratio are both close to 95%. The cycle-to-cycle comparison also shows good shape repetition, indicating that PAN-DA/GR nanocomposites can be used for repetitive shape memory processes at a temperature beyond *T*_g_ and blow *T*_rDA_. The little deviation in these three cycles also means that the plasticity is suppressed under the temperature of 75 °C for the elastic shape memory experiment. As a result, the thermally distinct elasticity and plasticity can be probed in consecutive thermomechanical cycles as shown in Fig. 4c. Within each cycle, an elasticity-based shape memory cycle was achieved with the shape fixity and shape recovery ratio both above 96%, followed by a plasticity cycle with the shape retention ratio approaching 90%. Here, the shape retention ratio is defined as *R*_ret_ = *ε*/*ε*_load_, where *ε* and *ε*_load_ corresponds to the strain amplitude at the specific plasticity temperature (135 °C) after and before load removal, respectively. Shape memory effect at highly deformation is still good (Fig. S4†) at different cycles. The above results clearly indicate that plasticity and elasticity can be triggered at two different temperatures. Moreover, no noticeable deterioration in the shape retention, shape fixity and recovery ratios was observed in the multiple thermomechanical cycles, which make it potential for achieving a cumulative plasticity and complex shape manipulation.

**Fig. 5:**
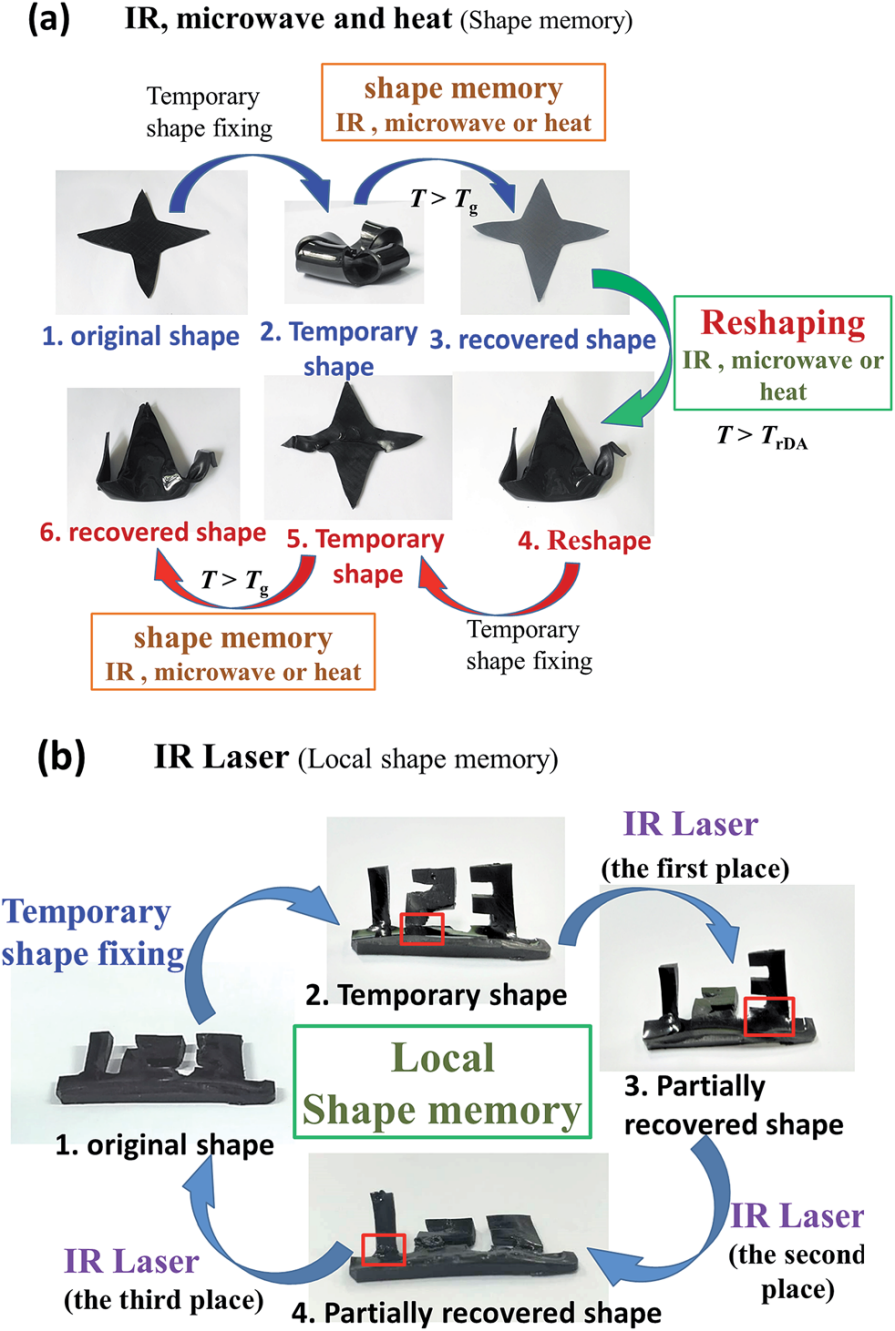
(a) Shape memory property of the PAN-DA/GR nanocomposites under different stimulus including microwave, IR light and heat. (b) Local shape memory property of PAN-DA/GR nanocomposites using IR laser with a power of 0.6 W cm^−2^.

In PAN-DA/GR nanocomposites, graphene bestow the materials with multiple-responsive ability, and thus the shape memory and self-healing/reshaping behaviours can be controlled by multiple approaches. Due to the thermally distinct elasticity and plasticity natures of PAN-DA/GR nanocomposites, complex 3D shape manipulations can be achieved *via* multiple approaches, as shown in Fig. 5. Fig. 5a shows that a starlike film can be folded into a temporary flower shape, which can recover to the original starlike shape by virtue of its elasticity above *T*_g_ under heat, IR or microwave irradiation. In the following, the same starlike film was folded plastically into a permanent shape of a bird at a temperature beyond *T*_rDA_, which later can be deformed into temporary shapes at a temperature beyond *T*_g_, and then recover under the stimulus of IR, microwave or heat. The above experiments demonstrate that the crosslinked PAN-DA/GR is not only a multiple-responsive shape memory materiel below *T*_rDA_, but also can be effectively plastically reshaped and reprocessed at temperature greater than *T*_rDA_.

Furthermore, regional shape memory property can also be controlled for PAN-DA/GR nanocomposite due to localized thermal effect induced by graphene in response to the localized IR irradiation. As shown in Fig. 5b, the numbers “1”, “2” and “3” shapes of the PAN-DA/GR-0.4 film was folded perpendicular to the horizontal plane as a temporary shape. Then the IR laser was irradiated at a targeted place (as denoted by red box in the figure) of the permanent shape in sequence, we observed the “2”, “3” and “1” recovered to its original shape one by one. The related shape memory video of the above experiments can be seen in the ESI.†

**Fig. 6:**
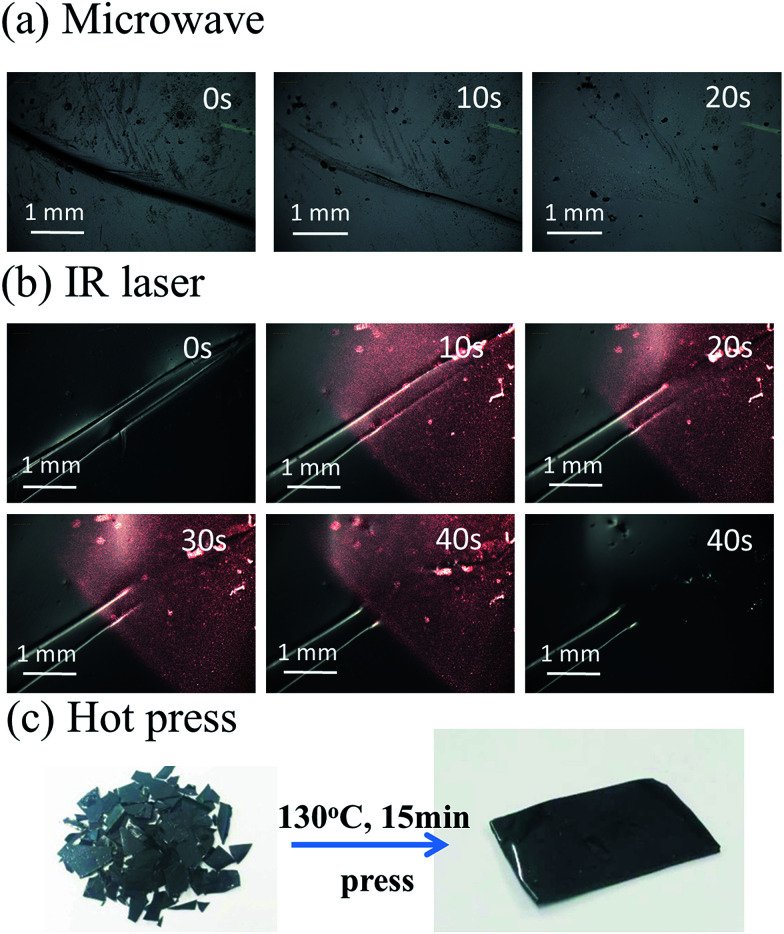
Optical microscopy images of the targeted healing or bulk recycling of the nanocomposite *via* multiple approaches, including (a) microwave, (b) IR laser (1 W cm^−2^) and (c) heat.

**Fig. 7:**
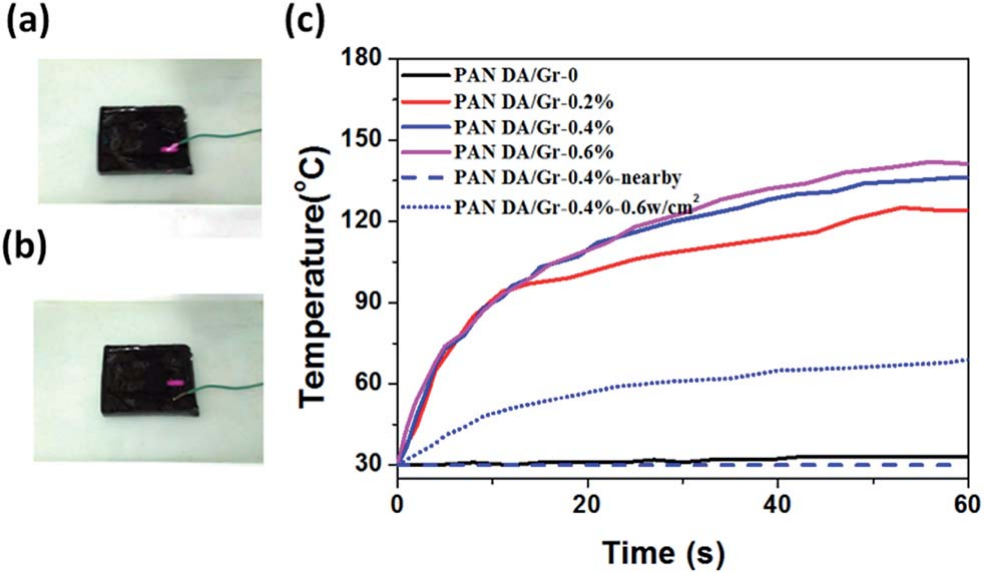
Experiments for measuring temperature of PAN-DA/GR-0.4 nanocomposite. (a) Experimental setup for measuring the temperature on the place where the IR laser is irradiated. (b) Experimental setup for temperature measurement on the sample surface where the IR laser is not irradiated. (c) The sample temperature as a function of the IR laser irradiation time for PAN-DA/GR nanocomposites with different graphene content. The power of IR laser for the experiment is 1 W cm^−2^ unless specified.

An obvious disadvantage for the thermoset SMPs is that the material cannot be reprocessed or remended once synthesized, which greatly imposes limitation on the service life of products. In this work, the unique property of the multiple-responsive PAN-DA/GR SMPs is able to self-heal and be recycled by multiple approaches as shown in Fig. 6. For example, the large crack on the PAN-DA/GR film can be easily and fast repaired in the microwave oven for a short time of 20 s (Fig. 6a). Furthermore, targeted healing/repairing can be realized for PAN-DA/GR by IR laser irradiation. As shown in Fig. 6b, the local crack on the surface of the film can be quickly healed within 40 s under IR laser irradiation. In fact, only the temperature of the local place where IR is irradiated will be affected as shown in Fig. 7. The temperature of the local position irradiated by IR increased much faster than that without IR irradiation. Moreover, a higher IR laser power also leaded to a faster increase in the temperature, as demonstrated on the PAN-DA/GR-0.4 sample. Thus complex local shape manipulation or healing can be achieved rapidly without affecting the integrity of the whole sample. Fig. 6c illustrates the hot compression molding of cracked pieces of PAN-DA/GR-0.4 sample. Typically, 15 minutes of processing time at 130 °C is sufficient to produce a recycled sample. The resulting polymers are completely reshaped and mended, which also clearly demonstrates the thermally recycling ability of this cross-linked SMPs. It is worth noting that all the above healing can be achieved in only a few minutes, or even seconds depending on the cracks. Such rapid and efficient healing can be ascribed to the fast disassociation of the DA network induced by the local heat from graphene absorbing IR lights/microwaves.

## Conclusions

In summary, a multiple stimuli-responsive thermally reversible crosslinked PAN-DA/GR composites is reported here by simultaneously incorporating Diels–Alder (DA) covalent bonds and multiple-responsive graphene into the PAN-DA matrix. DSC and *in situ* variable temperature ^13^C SSNMR results clearly confirmed the presence of DA/rDA reactions. Besides, a small amount of graphene (<1 wt%) has greatly increased the Young's modulus and tensile strength at break with slight compromise at the elongation ratio at break. Furthermore, the elasticity and plasticity natures of such SMPs were well demonstrated by multiple approaches including heat, IR and microwave irradiation, in addition to rapid self-healing and recycling properties. Indeed, the incorporation of Diels–Alder bonds has rendered the material solid state plasticity through topological network rearrangement, and thus leading to versatile shape adaptability. The cycle-to-cycle DMA experiments showed quite good repetition of shapes, indicating excellent shape memory behaviours for this nanocomposite. In fact, the graphene in proximity to the DA crosslinkages can act as an intrinsic localized thermal source, by effectively converting the absorbed external energies into heat, to trigger the glass transition for shape memory property and rDA reactions for self-healing/recycling. Local shape memory property and targeted self-healing can be achieved by IR laser irradiation. Our experimental findings here well demonstrated that the PAN-DA/ER nanocomposites could be a promising self-healing and shape memory material and find wide applications in various fields. Overall, the current work could also provide guidance for the design and fabrication of high-performance shape-memory polymers with rapid self-healing, recyclability and multiple-responsibility.

## Conflicts of interest

There are no conflicts to declare.

## Supplementary Material

RA-008-C7RA11484B-s001

RA-008-C7RA11484B-s002

## References

[cit1] Blaiszik B. J., Kramer S. L. B., Olugebefola S. C., Moore J. S., Sottos N. R., White S. R. (2010). Annu. Rev. Mater. Res..

[cit2] Chen Y., Kushner A. M., Williams G. A., Guan Z. (2012). Nat. Chem..

[cit3] Guan Z., Roland J. T., Bai J. Z., Ma S. X., McIntire T. M., Nguyen M. (2004). J. Am. Chem. Soc..

[cit4] Ju G., Guo F., Zhang Q., Kuehne A. J. C., Cui S., Cheng M., Shi F. (2017). Adv. Mater..

[cit5] de Espinosa L. M., Meesorn W., Moatsou D., Weder C. (2017). Chem. Rev..

[cit6] Zou W., Dong J., Luo Y., Zhao Q., Xie T. (2017). Adv. Mater..

[cit7] Zhao Z., Zhang K., Liu Y., Zhou J., Liu M. (2017). Adv. Mater..

[cit8] Zhao Q., Qi H. J., Xie T. (2015). Prog. Polym. Sci..

[cit9] Zheng N., Fang Z., Zou W., Zhao Q., Xie T. (2016). Angew. Chem., Int. Ed..

[cit10] Zhao R., Zhao T., Jiang X., Liu X., Shi D., Liu C., Yang S., Chen E.-Q. (2017). Adv. Mater..

[cit11] Zhao Q., Zou W., Luo Y., Xie T. (2016). Sci. Adv..

[cit12] Yang Z., Wang Q., Wang T. (2016). ACS Appl. Mater. Interfaces.

[cit13] Lendlein A., Kelch S. (2002). Angew. Chem., Int. Ed..

[cit14] Behl M., Lendlein A. (2007). Mater. Today.

[cit15] GhavamiNejad A., Stadler F. J., Vatankhah-Varnoosfaderani M., Hashmi S. (2014). Polym. Chem..

[cit16] Liu J., Tan C. S. Y., Yu Z., Li N., Abell C., Scherman O. A. (2017). Adv. Mater..

[cit17] Cuthbert T. J., Jadischke J. J., de Bruyn J. R., Ragogna P. J., Gillies E. R. (2017). Macromolecules.

[cit18] Ahner J., Micheel M., Geitner R., Schmitt M., Popp J., Dietzek B., Hager M. D. (2017). Macromolecules.

[cit19] Li C.-H., Wang C., Keplinger C., Zuo J.-L., Jin L., Sun Y., Zheng P., Cao Y., Lissel F., Linder C., You X.-Z., Bao Z. (2016). Nat. Chem..

[cit20] Neal J. A., Mozhdehi D., Guan Z. (2015). J. Am. Chem. Soc..

[cit21] Mukherjee S., Hill M. R., Sumerlin B. S. (2015). Soft Matter.

[cit22] Ji S., Cao W., Yu Y., Xu H. (2015). Adv. Mater..

[cit23] Ying H. Z., Zhang Y. F., Cheng J. J. (2014). Nat. Commun..

[cit24] Rekondo A., Martin R., Ruiz de Luzuriaga A., Cabanero G., Grande H. J., Odriozola I. (2014). Mater. Horiz..

[cit25] Yu S., Zhang R., Wu Q., Chen T., Sun P. (2013). Adv. Mater..

[cit26] Chen S., Wang F., Peng Y., Chen T., Wu Q., Sun P. (2015). Macromol. Rapid Commun..

[cit27] Zhang G., Zhao Q., Yang L., Zou W., Xi X., Xie T. (2016). ACS Macro Lett..

[cit28] Ninh C., Bettinger C. J. (2013). Biomacromolecules.

[cit29] Lakatos C., Czifrák K., Karger-Kocsis J., Daróczi L., Zsuga M., Kéki S. (2016). J. Appl. Polym. Sci..

[cit30] Huang L., Yi N., Wu Y., Zhang Y., Zhang Q., Huang Y., Ma Y., Chen Y. (2013). Adv. Mater..

[cit31] Wu S., Li J., Zhang G., Yao Y., Li G., Sun R., Wong C. (2017). ACS Appl. Mater. Interfaces.

[cit32] Boland C. S., Khan U., Ryan G., Barwich S., Charifou R., Harvey A., Backes C., Li Z., Ferreira M. S., Möbius M. E. (2016). Science.

[cit33] Kim H., Abdala A. A., Macosko C. W. (2010). Macromolecules.

[cit34] Wang M., Duan X., Xu Y., Duan X. (2016). ACS Nano.

[cit35] Cai C., Zhang Y., Zou X., Zhang R., Wang X., Wu Q., Sun P. (2017). RSC Adv..

[cit36] Schawe J. E. K., Hütter T., Heitz C., Alig I., Lellinger D. (2006). Thermochim. Acta.

[cit37] Zhang R., Mroue K. H., Ramamoorthy A. (2016). J. Magn. Reson..

[cit38] Xu H., Yu C., Wang S., Malyarchuk V., Xie T., Rogers J. A. (2013). Adv. Funct. Mater..

[cit39] Lendlein A., Behl M., Hiebl B., Wischke C. (2010). Expert Rev. Med. Devices.

[cit40] Scott T. F., Schneider A. D., Cook W. D., Bowman C. N. (2005). Science.

[cit41] Kloxin C. J., Scott T. F., Park H. Y., Bowman C. N. (2011). Adv. Mater..

[cit42] Montarnal D., Capelot M., Tournilhac F., Leibler L. (2011). Science.

[cit43] Röttger M., Domenech T., van der Weegen R., Breuillac A., Nicolaÿ R., Leibler L. (2017). Science.

