# Supplementary material for: Multiple-responsive shape memory polyacrylonitrile/graphene nanocomposites with rapid self-healing and recycling properties
Source: RSC Adv. 2018 Jan 3;8(3):1225–31. doi: 10.1039/c7ra11484b (PMC9076983; doi:10.1039/c7ra11484b)
Supplement: RA-008-C7RA11484B-s002 [file RA-008-C7RA11484B-s002.pdf]

Electronic Supplementary Material (ESI) for RSC Advances.
This journal is © The Royal Society of Chemistry 2017

Supporting Information

Multiple-Responsive Shape Memory Polyacrylonitrile/ Graphene Nanocomposites with Rapid Self-Healing and Recycling Properties

*Chenting Cai,[a] Yue Zhang,[b] Mei Li,[a] Yan Chen,[a] Rongchun Zhang,[c] Xiaoliang Wang,[b] Qiang Wu,[a] Tiehong Chen,[d] and Pingchuan Sun,[a,c]**

[a]Key Laboratory of Functional Polymer Materials of Ministry of Education and College of Chemistry, Collaborative Innovation Center of Chemical Science and Engineering (Tianjin), Nankai University, Tianjin 300071, China

[b]Department of Polymer Science and Engineering, Nanjing University, Nanjing 210093, China

[c]State Key Laboratory of Medicinal Chemical Biology, Nankai University, Tianjin, 300071, China

[d]Institute of New Catalytic Materials Science, School of Materials Science and Engineering, Key Laboratory of Advanced Energy Materials Chemistry (MOE), Nankai University, Tianjin 300350, China

Experimental

1. Sample Preparation

1.1 Materials. 1,1'-(methylenedi-4,1-phenylene) bismaleimide (BM, >96%) was purchased from TCI Chemicals Co., Ltd. (Tianjin, China), while Furfurylmercaptan (>99%) and triethylamine (Et_3N, >99%) were bought from Sigma Aldrich (Shandong, China). N-methyl-2-pyrrolidone (NMP) and tetrahydrofuran (THF) was obtained from Concord Technology Co.， Ltd. (Tianjin, China). Reduced graphene oxide (0.4mg/ml, dispersed in NMP solvent) was commercial available from Nanjing XFNANO Materials Tech Co.， Ltd. All chemicals were used as received without any further treatment. Acrylonitrile (AN) was purchased from Liyuan Chemical Reagent Co., Ltd. （Tianjin，China）, while glycidyl methacrylate (GMA), methyl butyl acrylate (BA) and azodiisobutyronitrile (AIBN) were obtained from J&K Scientific., Ltd. (Shanghai, China). AN, BA and GMA were distilled before used, and AIBN was used after recrystallization.

1.2 Synthesis of liner PAN-Furan. To obtain DA-crosslinked PAN-DA/GR, furan grafted liner polymer (PAN-Furan) was first synthesized, and then crosslinked by BM. As shown in Scheme S1, linear epoxy grafted PAN (PAN-epoxy) was obtained by the free radical polymerizations between AN, BA and GMA monomers in DMF solution, using AIBN as the initiator. AN (44g), BA (9.6g), GMA (6g) and AIBN (1.3g) were added via drops over a period of 3 h into DMF (60g) in the flask at a temperature of 80 °C. The mixture was further kept at 80 °C to obtain the viscous PAN-epoxy solution, which was later poured into methanol. As a result, the PAN-epoxy would precipitate from the solvent, and later was dried in a vacuum oven for 24 h at 50 °C. The dried PAN-epoxy solids (30 g) were further dissolved in DMF solvent (60g) in together with furfurylmercaptan (2.8 g) and triethylamine (Et3N, 0.1 g). After stirring at 70 °C for 16 hrs, the mixture was precipitated in methanol (500 mL) and dried in a vacuum oven for 24 h at 50 °C. The chemical structures of PAN-epoxy and PAN-Furan were confirmed by solution ^1H NMR experiments, as shown in Figure S1 and S2.

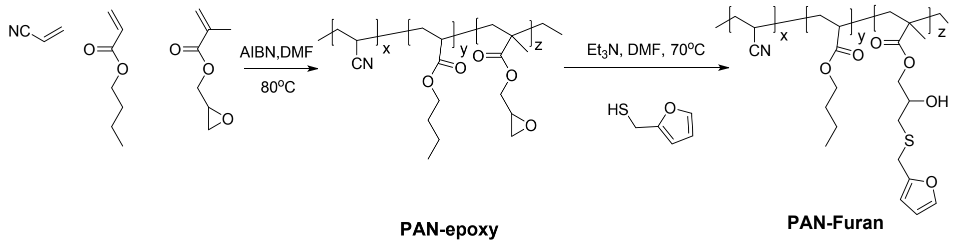

Scheme S1. Synthesis of PAN-Furan.

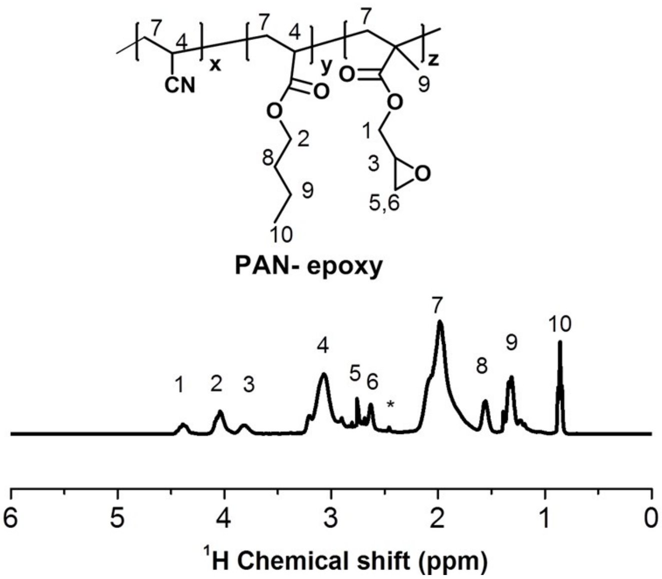

Figure S1. ^{1}H NMR spectrum of PAN-epoxy in DMSO-D6. "*" denotes the proton signals of DMSO-D6 (δ_{iso}=2.51 ppm) .

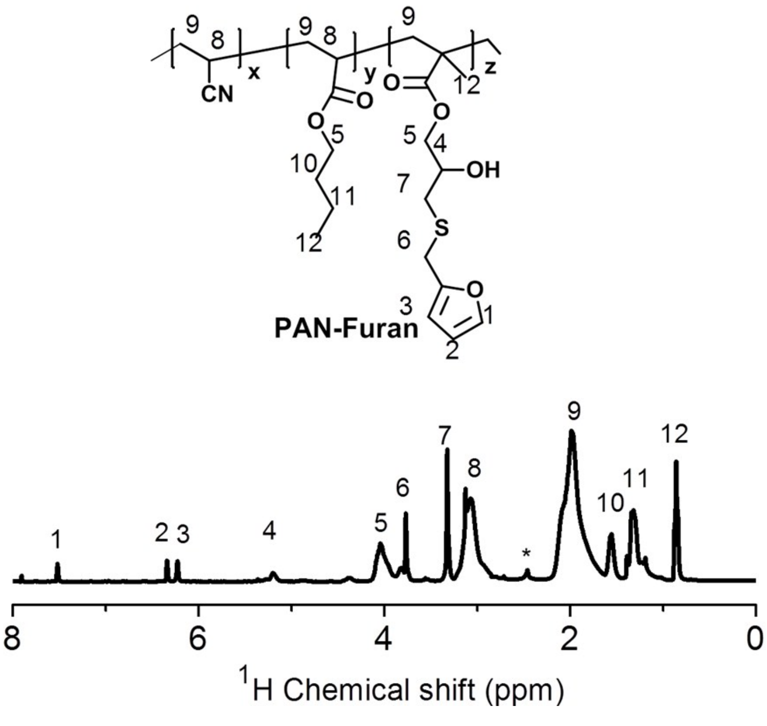

Figure S2. ^{1}H NMR spectrum of PAN-Furan in DMSO-D6. "*" denotes the proton signals of DMSO-D6 (δ_{iso}= 2.51 ppm).

1.3 Preparation of PAN-DA/GR. The schematic procedures for the preparation of PAN-DA/GR

nanocomposites were shown in Scheme S2. PAN-furan was added into the NMP solvent with well dispersed graphene, leading to a 30wt % NMP solution. After ultrasonic treatment for 30 mins, the resulting mixture was poured onto a teflon mold, and dried under vacuum at 65°C for three days. Four samples, corresponding to a graphene weight fraction of 0, 0.25%, 0.5% and 0.75%, were prepared and named as PAN-DA/GR, PAN-DA/GR-0.25, PAN-DA/GR-0.5, PAN-DA/GR-0.75, respectively.

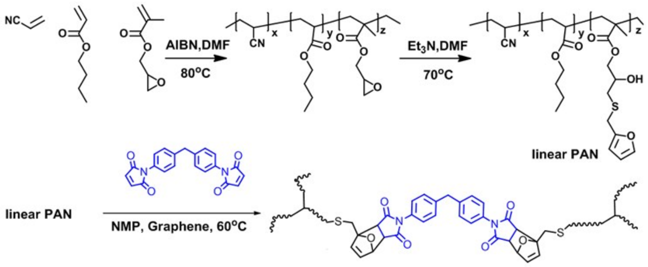

Scheme S2. Synthesis routes for thermal reversible crosslinked PAN-DA.

2. Characterization

2.1 Differential Scanning Calorimetry (DSC). DSC measurements were carried out on a Mettler-Toledo DSC1 differential scanning calorimeter using a nitrogen atmosphere with a cooling and heating rate of 10°C min^{-1}. About 10 mg samples were encapsulated in a 40μL aluminum pan for DSC measurements. TOPEM, a new stochastic temperature-modulated DSC technique introduced by Mettler-Toledo in late 2005, was also perform to distinguish the glass transition process and rDA reaction of the polymeric materials. The temperature scan was set from 0°C to 150°C to cover the glass transition and rDA reaction intervals. The underlying heating rate was set as 1°C/min, and the amplitude of temperature pulse was set as 1°C. The switching time range, which limits the duration of the pulses, was set as 15-30s.

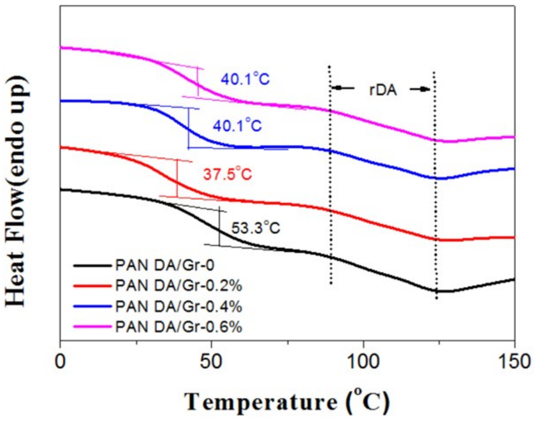

Figure S3. DSC traces of PAN-DA/GR with different graphene content.

2.2 NMR experiments. Solution ^{1}H NMR experiments were performed on a Bruker AVANCE III NMR spectrometer with a proton resonance frequency of 400.13 MHz. The samples were dissolved in deuterated DMSO.

High-field Solid-state NMR (SSNMR) experiments were performed on a Varian InfinityPlus-400 wide-bore (89mm) NMR spectrometer operating at a ^{1}H frequency of 399.7 MHz and ^{13}C frequency of 100.5 MHz, respectively. A conventional 4mm double-resonance HXCP/MAS NMR probe was used, and samples were placed in a 4 mm zirconia PENCIL rotor with a well-sealed cap and spacer. The magic angle spinning (MAS) was automatically controlled at 10 kHz within ± 2 Hz with a magic angle spinning (MAS) speed controller. In-situ variable-temperature CP-NOE experiments,[2] which are aimed to simultaneously enhance the signals of both rigid and mobile components, were carried out in the temperature range of 25~130 °C with a Varian Model-L950 temperature controller. Heteronuclear decoupling during the acquisition period was achieved by SPINAL-64 irradiation[3] with a radiofrequency strength of ~80 kHz. A temperature equilibration period of 10 min was implemented before NMR spectra were acquired at each measurement temperature. The recycle delay was set to 4 s.

2.3 Mechanical testing.

Stress-strain curves were measured on an UTM6103 mechanical testing instrument (Shenzhen Suns Technology Stock Co., LTD., China) in tensile mode at a room temperature of 20 °C. The samples were cut into a shape of 12 mm x 2 mm x 1 mm, and the strain rate is 5 mm/min. The stress relaxation experiments were performed on a Dynamic Mechanical Analysis machine (DMA Q800) in a constant-strain mode. Samples were cut into rectangular shapes (30mm*5mm*1mm).

2.4 Shape Memory Property

For quantitative shape memory demonstrations, a temporary shape was achieved at 75 °C (above T_g) and then cooled to 20 °C to fix the temporary shape. The fixed shape was then placed back in heating stage to recover. In plastic reshape and shape memory test, a plastic reshape was obtained at 135 °C (above T_{rDA}) and shape memory test was then placed. The shape fixity and shape recovery ratio (R_{fix} and R_{ret}) are calculated using the method in reference S1.

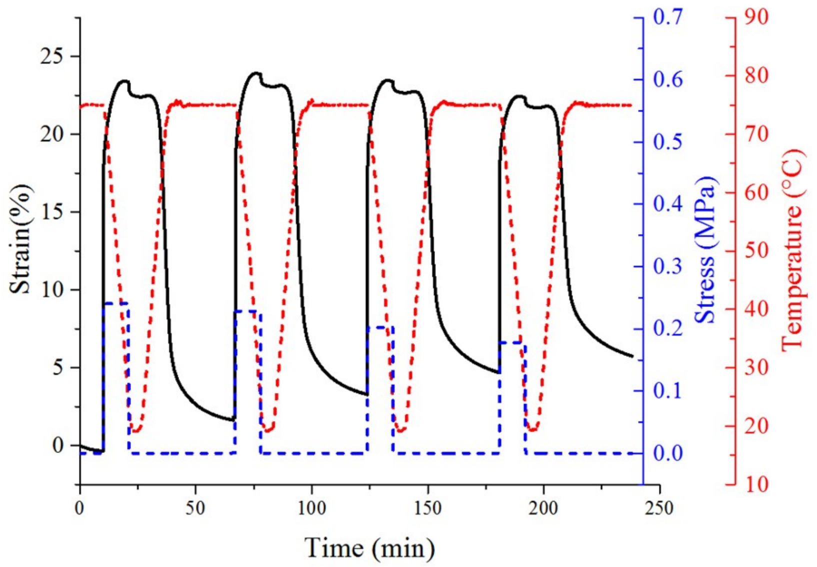

Figure S4. Thermomechanical characterization of shape memory cycles at large strain. Cycle 1: R_{fix}=95.9%, R_{ret}=93%; Cycle 2: R_{fix}=96.6%, R_{ret}=92.7%; Cycle 3: R_{fix}=96.7%, R_{ret}=93%; Cycle 4: R_{fix}=95.9%, R_{ret}=90.3%.

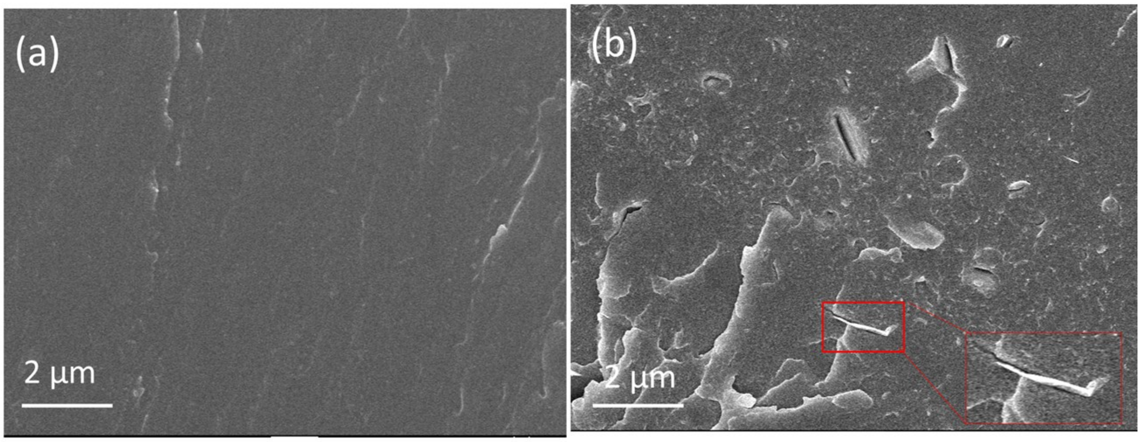

Figure S5. SEM images of the cryogenically fractured surfaces of (a)PAN-DA/GR-0 and (b) PAN-DA/GR-0.4 samples. In PAN-DA/GR-0.4 SEM images, the large hole defects indicated by the red circles are ascribed to the separation of graphene from another fractured surface, which also well indicates the well dispersion of graphene in the polymer matrix.

Reference

S1. Tao Xie， Tunable polymer muti-shape memory effect, Nature, 2010, 464, 267.
